# Supplementary material for: Observation of geometry-dependent skin effect in non-Hermitian phononic crystals with exceptional points
Source: Nat Commun. 2023 Jul 29;14:4569. doi: 10.1038/s41467-023-40236-7 (PMC10387049; doi:10.1038/s41467-023-40236-7)
Supplement: Supplementary file 1 — Supplementary Information [file 41467_2023_40236_MOESM1_ESM.pdf]

Supplementary Information for
Observation of geometry-dependent skin effect in non-Hermitian phononic crystals with exceptional points

Qiuyan Zhou[1†], Jien Wu[2†], Zhenhang Pu[1], Jiuyang Lu[1,2], Xueqin Huang[2], Weiyin Deng[1,2*], Manzhu Ke[1*], and Zhengyou Liu[1,3*]

[1]Key Laboratory of Artificial Micro- and Nanostructures of Ministry of Education and School of Physics and Technology, Wuhan University, Wuhan 430072, China
[2]School of Physics and Optoelectronics, South China University of Technology, Guangzhou, Guangdong 510640, China
[3]Institute for Advanced Studies, Wuhan University, Wuhan 430072, China

†These authors contributed equally to this work.
*Corresponding author. Email: dengwy@whu.edu.cn; mzke@whu.edu.cn; zyliu@whu.edu.cn

S-I. Analyzing the tight-binding model

S-II. Designed loss in experiments

S-III. Detailed spectra of the PC

S-IV. Observation of the bulk Fermi arc in the 2D PC

S-V. Band dispersions near the EPs for different temperatures

S-VI. GDSE in the 1D PC ribbons

S-VII. No skin effect in the finite-size PC under square-shaped geometry

S-VIII. Pressure responses for different distances away from source

S-I. Analyzing the tight-binding model

A. EPs in the non-Hermitian model

In this subsection, we investigate the Bloch Hamiltonians in Hermitian and non-Hermitian cases, and show how a Dirac point splits into a pair of EPs connected by a bulk Fermi arc. As shown in Fig. 1a of the main text, the Hamiltonian of the tight-binding lattice in Hermitian case ($\gamma = 0$) has the form

$$H_0 = d_x\sigma_x + d_y\sigma_y, \tag{S1}$$

where $d_x = (t_1 + t_2)\cos k_x + 2t_y \cos k_y$, $d_y = (t_1 - t_2)\sin k_x$. The distance $a/\sqrt{2}$ between sublattices is set to unity for simplicity. $\mathbf{k} = (k_x, k_y)$ is the wavevector. The corresponding eigenvalue is

$$E_0 = \pm\sqrt{d_x^2 + d_y^2}. \tag{S2}$$

The bulk band dispersion near a Dirac point is shown in Fig. S1a, in which the upper and lower bands are degenerate at a Dirac point. The positions of the Dirac points can be exactly solved by $d_x = 0$ and $d_y = 0$, and obtained at $\mathbf{k}_{0,\pm} = \left(0, \pm\arccos\left(\frac{t_1+t_2}{2t_y}\right)\right)$ in the first BZ.

We then consider the loss interaction γ on hopping t_1. As shown in Fig. 1a of the main text, the non-Hermitian Bloch Hamiltonian becomes

$$H = (d_x + i\gamma\cos k_x)\sigma_x + (d_y + i\gamma\sin k_x)\sigma_y, \tag{S3}$$

with complex eigenvalues

$$E = \pm\sqrt{(d_x^2 + d_y^2 - \gamma^2) + 2i\gamma(d_x\cos k_x + d_y \sin k_x)}. \tag{S4}$$

For further analysis, we write the eigenvalues of Eq. (S4) as argument form

$$E = \pm\sqrt{Re^{i\theta}} = \pm\sqrt{R}\left(\cos\frac{\theta}{2} + i\sin\frac{\theta}{2}\right), \tag{S5}$$

where $R = \sqrt{P^2 + Q^2}$ with $P = R\cos\theta = d_x^2 + d_y^2 - \gamma^2$ and $Q = R\sin\theta = 2\gamma(d_x\cos k_x + d_y\sin k_x)$. The real part degeneracy of the band dispersion occurs when $\cos\theta/2 = 0$, i.e., $\theta = \pm\pi$, which means that $P \leq 0$ and $Q = 0$, i.e.,

$$d_x^2 + d_y^2 - \gamma^2 \leq 0, \tag{S6}$$

$$2\gamma(d_x\cos k_x + d_y\sin k_x) = 0. \tag{S7}$$

If we limit the range for wave vector in the first Brillouin zone, it is easy to obtain the solution of Eqs. S6 and S7 as

$$-\arctan\frac{\gamma}{t_2-t_1} \leq k_x \leq \arctan\frac{\gamma}{t_2-t_1}, \tag{S8}$$

$$k_y = \pm\arccos\left(-\frac{t_1-t_2}{2t_y}\sin k_x \tan k_x - \frac{t_1+t_2}{2t_y}\cos k_x\right), \tag{S9}$$

which indicates the bulk Fermi arcs, as shown in the left panel of Fig. S1b. When taking the limit of Eq. (S8), we can obtain the EPs solved by $P = 0$ and $Q = 0$, locating at the ends of the bulk Fermi arc. The second-order EPs are stable in two dimensions. Figure S1b shows a bulk Fermi arc is terminated by a pair of EPs, where the red (blue) sphere denotes the EPs with topological charge $+1$ (-1). So the bulk Fermi arc can reveal the existence of EPs.

To summarize, by introducing hopping loss in our model, a Dirac point will split into a pair of second-order EPs connected by a bulk Fermi arc. There are four EPs in the first BZ at positions $\mathbf{k}_{1,\pm} = (\pm k_{x0}, k_{y0})$, $\mathbf{k}_{2,\pm} = (\pm k_{x0}, -k_{y0})$ with topological charges $+1$, -1, $+1$, -1, respectively. Here, $k_{x0} = \arctan\frac{\gamma}{t_2-t_1}$, and $k_{y0} = \arccos\left(\frac{\gamma^2+t_1^2-t_2^2}{2t_y\sqrt{(t_1-t_2)^2+\gamma^2}}\right)$. Obviously, they will turn to a pair of Dirac points at positions $\mathbf{k}_{0,\pm}$ in the absence of hopping loss with $\gamma = 0$.

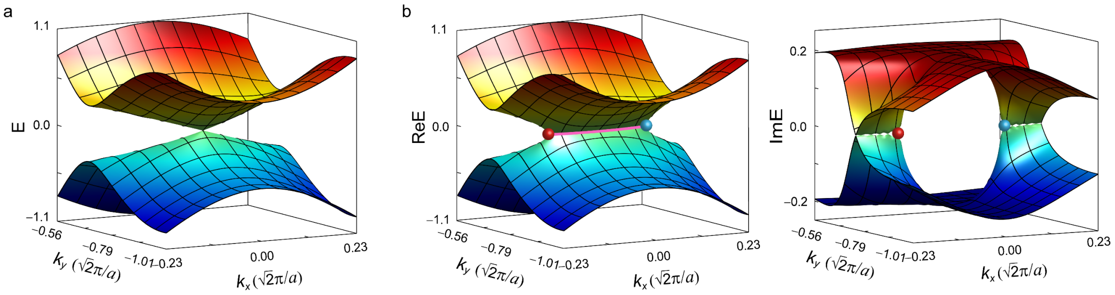

Fig. S1 | Bulk band dispersion near EPs. a, Bulk band dispersion of Hermitian case with $\gamma = 0$ near Dirac point. **b**, Bulk band dispersion of non-Hermitian case with $\gamma = 0.2$. Left panel: real part of the band dispersion. A pair of EPs split by a Dirac point are connected by a bulk Fermi arc (magenta line), which have different topological charges $+1$ (red sphere) and -1 (blue sphere). Right panel: imaginary part of the band

dispersion. The parameters are chosen as $t_1 = t_y = -1$ and $t_2 = -0.5$.

B. Detailed spectra of the tight-binding model

Here, we provide the detailed spectra of the tight-binding model under periodic boundary conditions in Fig. S2, including the dispersion curves (real and imaginary parts) along high symmetry lines, as a function of k_x for fixed k_y, and as a function of k_y for fixed k_x. These results clearly show the exceptional points and bulk Fermi arcs existed in the tight-binding model.

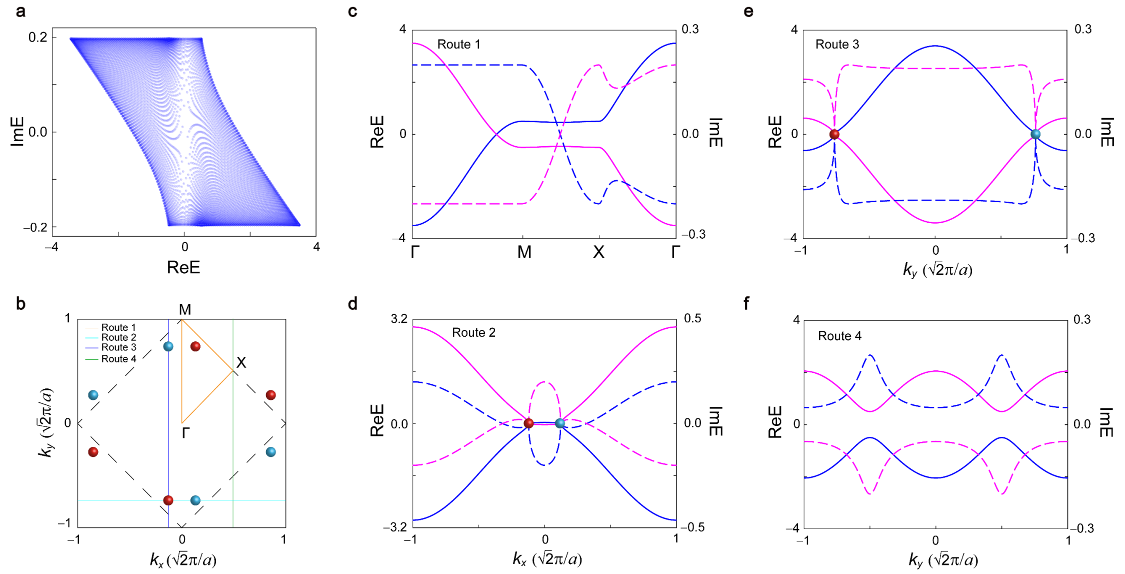

Fig. S2 | Bulk band dispersions of the tight-binding model for different routes. a, Spectral area under periodic boundaries. **b**, Schematic of routes in momentum space. The black dashed line encloses the first Brillouin zone. Red (light blue) spheres denote the position of exceptional points with $+1$ (-1) topological charge. Yellow, cyan, blue and green lines represent the chosen routes 1-4, respectively. **c**-**f**, Dispersion curves containing both real (solid lines) and imaginary parts (dashed lines) along the routes 1-4, respectively. Degeneracy of the bulk Fermi arc emerges in **c**, while degeneracies of exceptional points occur in **d** and **e**. No degeneracy occurs in **f**.

C. Calculation of topological charge of EPs

In this subsection, we provide the method to calculate the topological charge of

EPs. The topological charge of EP can be described by the discriminant number calculated by

$$\nu(\mathbf{k}_{EP}) = \frac{1}{2\pi i}\oint_{\Gamma_{EP}} d\mathbf{k}\cdot\nabla_{\mathbf{k}} \ln\det[H(\mathbf{k}) - E(\mathbf{k}_{EP})], \tag{S10}$$

where Γ_{EP} is a closed counterclockwise route enclosing the reference EP. This topological invariant is actually the spectral winding number. We can discretize the integral for numerical calculation. Before that, we give tacit consent to choose a circle as the closed route with radius r, so that we can parameterize the route with radius r and angle λ. The coordinate of the points on the circle route can be expressed by these parameters, i.e., $k_x = k_{EP,x} + r\cos\lambda$ and $k_y = k_{EP,y} + r\sin\lambda$, where $\mathbf{k}_{EP} = (k_{EP,x}, k_{EP,y})$ is the position of reference EP, and λ takes from 0 to 2π. Now, the integral with $\mathbf{k}$ can be changed

$$\nu(\mathbf{k}_{EP}) = \frac{1}{2\pi i}\int_0^{2\pi} d\ln\det[H(\lambda) - E_{EP}] = \frac{1}{2\pi i}\int_0^{2\pi}\frac{F'(\lambda)}{F(\lambda)}d\lambda, \tag{S11}$$

where $F(\lambda) = \det[H(\lambda) - E_{EP}]$. For numerical calculation, we should discretize the integral from 0 to 2π into the sum of several points

$$\nu(\mathbf{k}_{EP}) = \frac{1}{2\pi i}\sum_n \frac{F_{n+1} - F_n}{F_n}, \tag{S12}$$

where n is the index of the points on the circle route. This method also is applied to calculate the topological charges of EPs in the PC.

D. Relation between the EPs and the GDSE

The relation between EP and GDSE is that the EP with nonzero topological charge can guarantee the emergence of the GDSE, but it is not vice versa. A brief outline of the relation is illustrated in Fig. S3a. Firstly (Step I), the EPs host nonzero topological charges. Secondly (Step II), the nonzero topological charges lead to the nonzero spectral winding numbers along some straight lines (directions) in the first Brillouin zone, which is equivalent to nonzero spectral area. Thirdly (Step III), the ribbons under open boundaries along the direction with nonzero spectral winding numbers host the skin effect, while those with zero winding number do not, giving rise to the GDSE in the 1D ribbons. Finally (Step IV), the GDSE in the 1D ribbons can directly result in the GDSE in the finite-size samples with fully open boundaries. It is noted that the EPs are

not the must for the topological charges (Step I is single arrow), while the others are equivalent to each other (Step II-IV are double arrows).

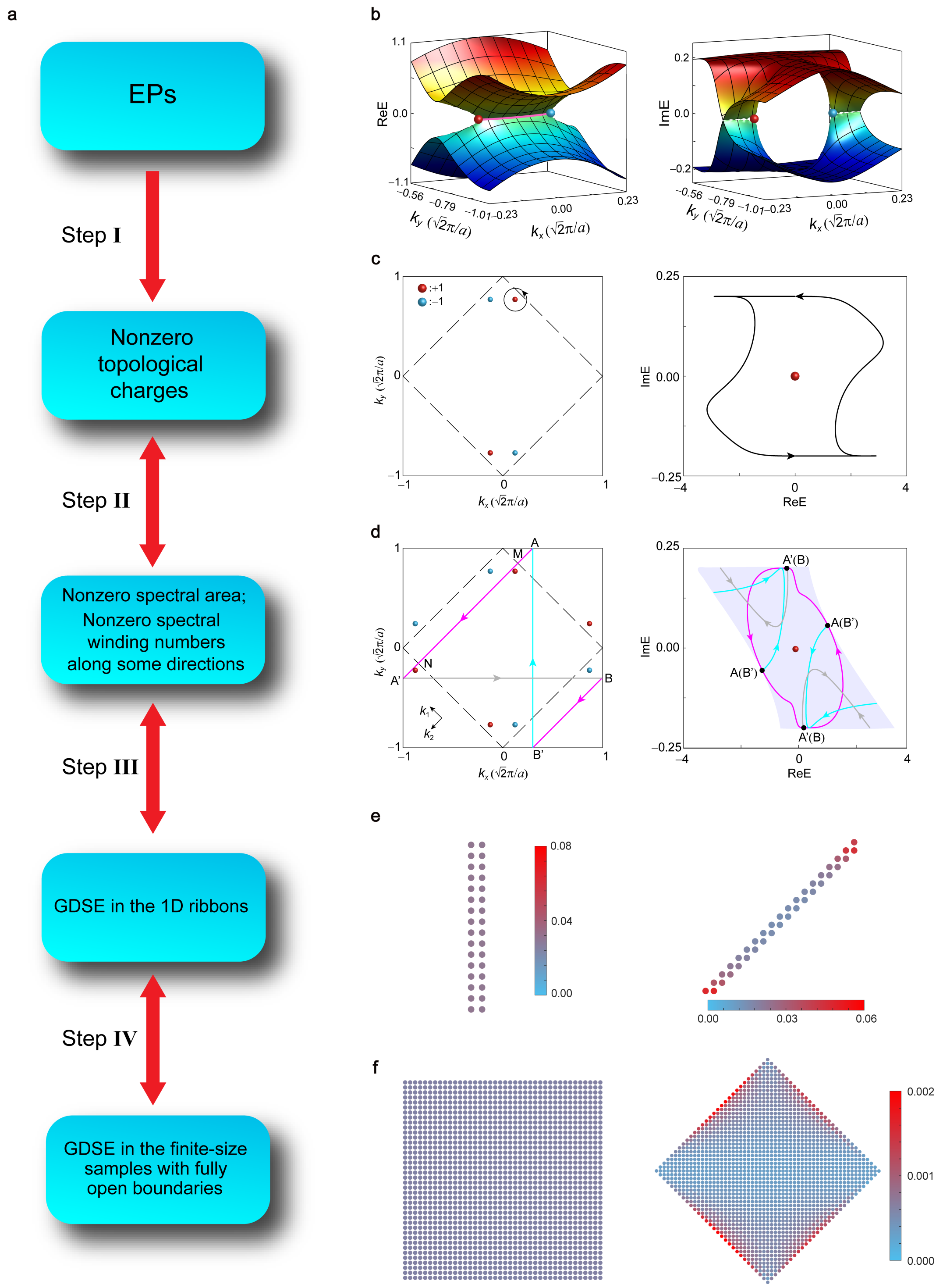

Fig. S3 | Relation between EPs and GDSE. a, A brief outline of the relation. The double arrows denote the equivalency, while the single arrow means a sufficient but not necessary condition. **b**, Bulk band dispersion near EPs. **c**, Left panel: A schematic

of momentum space. The area enclosed by dash lines is the first Brillouin zone. Red (blue) spheres denote EPs with $+1$ (-1) topological charge. Right panel: Spectral loop on the complex plane plotted along the anticlockwise direction of the black circle route around the EP in the left panel. **d**, Left panel: Three chosen routes $\mathrm{AA'BB'}$, $\mathrm{A'B}$ and $\mathrm{B'A}$ are denoted by magenta, gray and cyan lines, respectively. Right panel: Complex spectra of these three routes in the left panel. The blue background denotes the spectral area under periodic boundaries. **e**, Spatial distributions of $W(j)$ in the ribbons under square- (left panel) and diamond- (right panel) stripe geometries. **f**, Spatial distributions of $W(j)$ in the finite-size samples under square- (left panel) and diamond- (right panel) shaped geometries.

Our lattice model is a concrete example to exhibit this relation. Figure S3b displays a pair of EPs connected by the bulk Fermi arc. The spectra loop winds the EP (red sphere) in an anticlockwise direction (arrow), indicating $+1$ topological charge of EP, as shown in Fig. S3c. Figure S3d demonstrates that the $+1$ charge of EP leads to nonzero spectral winding numbers along the k_2 direction. As shown in the left panel of Fig. S3d, when choosing the closed route $\mathrm{A} \to \mathrm{A'} \to \mathrm{B} \to \mathrm{B'} \to \mathrm{A}$ encloses two EPs with $+1$ charge, the spectral winding number is the same to the sum of the topological charge of these two EPs. The winding number of this route is also equal to the sum of winding numbers for straight line loops $\mathrm{AA'BB'}$ (magenta), $\mathrm{A'B}$ (gray) and $\mathrm{B'A}$ (cyan). Due to the mirror symmetry M_x satisfying $M_x H(k_x, k_y) M_x^{-1} = H(-k_x, k_y)$, the spectral winding number of route $\mathrm{A'B}$ satisfies

$$\begin{aligned}\nu(E_{EP}) &= \frac{1}{2\pi i}\oint_{L_{\mathrm{A'B}}} dk_x \partial_{k_x} \ln \det[H(k_x, k_y)] \\ &= \frac{1}{2\pi i}\oint_{L_{\mathrm{A'B}}} dk_x \partial_{-k_x} \ln \det[H(-k_x, k_y)] \\ &= -\nu(E_{EP}) = 0, \end{aligned} \quad \text{(S13)}$$

where $L_{\mathrm{A'B}}$ indicates the integral along route $\mathrm{A'B}$. Similarly, due to the mirror symmetry M_y, the spectral winding number of line $\mathrm{B'A}$ is also zero. So the winding number of straight line $\mathrm{AA'BB'}$ is equal to $+2$, attributing to the charges of these two EPs. Since the straight line $\mathrm{AA'BB'}$ is the result of repeating line MN twice, the

winding number of line MN is equal to $+1$, half of the winding number of $\mathrm{AA'BB'}$. Correspondingly, the complex spectrum of line $\mathrm{AA'BB'}$ (magenta) goes anticlockwise around the energy of EP twice, forming the spectral loop, while those of lines $\mathrm{A'B}$ (gray) and $\mathrm{B'A}$ (cyan) form spectral lines, as shown in the right panel of Fig. S3d. Therefore, the winding numbers along the k_x and k_y directions are zero, but those along the k_2 direction are nonzero. By a similar way, one can see that the nonzero winding numbers also exist along the k_1 direction. These results make the ribbons under open boundaries along the x and y directions have no skin effect, and the ribbons under open boundaries along the 1 and 2 directions exhibit the skin effect, giving rise to the GDSE in the 1D ribbons, as shown in Fig. S3e. Naturally, the skin effect emerges in the finite-size sample under the same open boundaries to the ones in the ribbons. So the skin effect disappears in the sample under square-shaped geometry, and appears under diamond-shaped geometry, thus is the GDSE, as shown in Fig. S3f. Consequently, the EP with nonzero topological charge can ensure the GDSE.

E. Symmetry analysis

In this subsection, we make analysis for the symmetries of our bulk Hamiltonian shown in Eq. (1) of the main text. First of all, the Hamiltonian has spinless anomalous time-reversal symmetry (TRS), which satisfies $\mathcal{T}^{-1}H(\mathbf{k})\mathcal{T} = H(-\mathbf{k})$ and the unitary operator $\mathcal{T}$ satisfies $\mathcal{T}^2 = +1$. Due to the existence of anomalous TRS, the current functional in our system along any direction will be zero so that the skin effect appearing in our system is classified as generalized reciprocal skin effect [1]. Not only that, the Hamiltonian also has two mirrors parallel to x axis and y axis, respectively, i.e, $M_y H(k_x, k_y) M_y^{-1} = H(k_x, -k_y)$ and $M_x H(k_x, k_y) M_x^{-1} = H(-k_x, k_y)$. Due to the existence of these two mirrors, the spectral winding number of the straight lines perpendicular to them (x axis and y axis) will always be zero, so that we can confirm that the open boundaries perpendicular to x axis and y axis will not have skin effect in our system. For this reason, no skin effect emerges in the 1D ribbon under square-stripe geometry and finite-size sample under square-shaped geometry. Since the mirror

symmetries still exist in the PC, this method of judging open boundaries without skin effect also can be applied to our PC system and the result perfectly matches that in the tight-binding model.

F. Spectral winding numbers of straight lines

There is a general rule to evaluate the geometry dependence on the skin effect. Whether the skin effect emerges at the open boundaries vertical to the n direction or not, depends on the winding number of the straight lines along the k_n direction, which is defined as

$$\nu(E_r) = \frac{1}{2\pi i} \oint_L d\mathbf{k} \cdot \nabla_{\mathbf{k}} \ln \det[H(\mathbf{k}) - E_r], \tag{S14}$$

where L is the closed straight line along the k_n direction in the first Brillouin zone, and E_r is the energy of reference, a reference point on the complex plane of energy. In addition, some spatial symmetries may guarantee the winding numbers along the related directions to be zero. Nonzero winding number describes the spectral winding around the E_r on the complex plane, which is known as the point gap [2,3]. As a result, in the 1D system, the skin effect can also be predicted by the spectral loop of the complex energy. For example, we choose four straight lines to calculate their spectral loops. As shown in Fig. S4a, routes 1 and 2 match the ribbons under square-stripe geometry (Fig. S6), while routes 3 and 4 are related to the ribbons under diamond-stripe geometry (Fig. S7). Bulk energies of routes 1-4 in complex plane are shown in Figs. S4b and S4c. The routes 3 and 4 form the spectral loops, giving rise to nonzero spectral winding numbers for the reference energy E_r shown in Figs. S4b and S4c and indicating the existence of skin effect in the ribbons under diamond-stripe geometry. In contrast, the routes 1 and 2 do not form the spectral loops, leading to zero winding number and no skin effect in the ribbons under square-stripe geometry.

To further verify the general rule, we investigate the geometry-dependent skin effect in a parallelogram-shaped sample, in which the slope of the sloping open boundaries is 2, as shown in Fig. S5. The chosen route $\mathrm{A} \to \mathrm{B} \to \mathrm{C} \to \mathrm{D} \to \mathrm{A}$ (straight lines in magenta) vertical to the direction of the sloping open boundaries is shown in

Fig. S5a, which forms a closed loop in the first Brillouin zone. As shown in Fig. S5b, the solid (dashed) line is the spectrum of the upper (lower) band. The spectral winding number is nonzero, leading to skin effect on the sloping open boundaries of the parallelogram-shaped sample (Fig. S5c). The winding number along the k_y direction is zero, same to the case in the square-shaped sample, so no skin effect emerges at the horizontal open boundaries. Consequently, the geometry dependence on the skin effect can be generally evaluated by the spectral winding number along the corresponding direction.

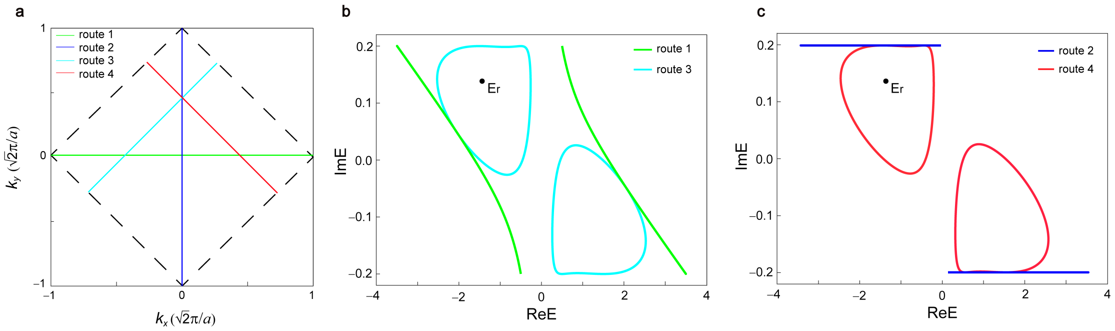

Fig. S4 | Spectral winding numbers of straight lines in the first BZ. a, Chosen straight lines in momentum space. The area enclosed by black dashed lines is the first BZ and the solid lines with different colors are the routes of straight line. **b**, Spectral lines of routes 1 (green solid line) and 3 (cyan solid line). **c**, Spectral lines of routes 2 (purple line) and 4 (red line). E_r is the reference energy. The parameters used above are the same as Fig. 1 of the main text.

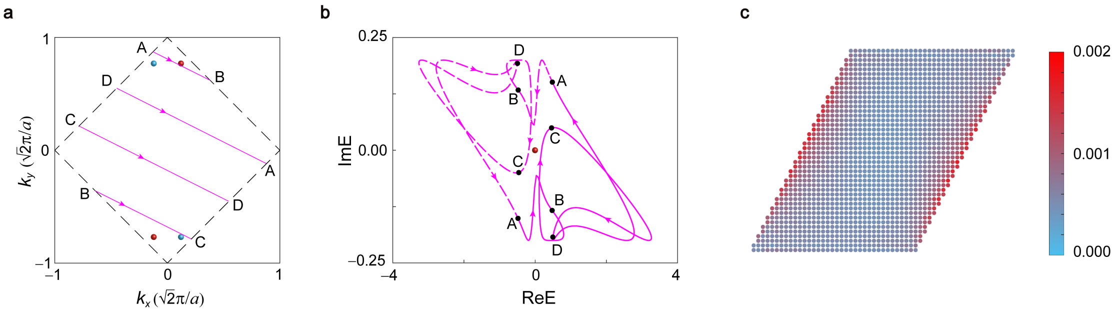

Fig. S5 | Skin effect under parallelogram-shaped geometry with sloping open boundaries possessing slope 2. a, Chosen route $A \rightarrow B \rightarrow C \rightarrow D \rightarrow A$ (magenta lines) vertical to the direction of the sloping open boundaries in the first Brillouin zone

(dashed lines). The arrows denote the direction of the route. **b**, Complex spectra of chosen route in **a**. Black points denote the energy of A-D points in **a**. Solid (dashed) line is the spectrum of the upper (lower) band. The winding number is $+1$ when the exceptional point (red sphere) is chosen as the energy of reference. **c**, Spatial distribution of eigenstates $W(j)$ under parallelogram-shaped geometry. The skin effect appears at the two sloping boundaries, and disappears at the two horizontal boundaries.

G. GDSE in the 1D ribbons

In order to demonstrate the existence of GDSE in the 1D ribbon, we investigate the ribbons under different shaped geometries, i.e., square-stripe and diamond-stripe geometries. The ribbons under square-stripe (diamond-stripe) geometry mean that their open boundaries are same to those along a fixed direction in the finite-size sample under square-shaped (diamond-shaped) geometry.

Ribbons under square-stripe geometry. As shown in Fig. S6a, one ribbon model has periodic boundaries along the x direction and open boundaries along the y direction. The projected band dispersions for Hermitian ($\gamma = 0$) and non-Hermitian ($\gamma = 0.2$) cases are plotted in Figs. S6b and S6c, respectively. In non-Hermitian case, states near zero energy are still bulk states similar to Hermitian one. For example, the square modulus of an eigenstate (green star) as the bulk state is shown Fig. S6d. We further calculate the spatial distribution of all eigenstates $W(j)$ for 1D ribbon, as shown in Fig. S6e, which is defined by the sum of the square modulus of the normalized right eigenstate at size j in the ribbon for all chosen k_x from -0.5 to 0.5 with interval 0.01. The spatial distribution $W(j)$ is almost uniform in the whole ribbon, indicating that no skin effect emerges in this ribbon. Figure S6f gives a schematic of the ribbon model along the other direction, in which there are periodic boundaries along the y direction and open boundaries along the x direction. Figures S6g and S6h are the corresponding projected band dispersions in Hermitian and non-Hermitian cases, respectively. The degenerate eigenstates denoted by gray dots are the trivial edge states induced by open boundaries, as shown in Fig. S6i. Despite existing the trivial edge

states, the spatial distribution $W(j)$ for the ribbon summing for k_y from -0.5 to 0.5 with interval 0.01 still locates uniformly in the ribbon (Fig. S6j). The edge states do not have influence on the skin effect. Consequently, there is no skin effect in the ribbons under square-stripe geometry.

Ribbons under diamond-stripe geometry. Figure S7a shows the schematic of the ribbons under diamond-stripe geometry. The green (red) wireframe represents the ribbon with periodic boundaries along the 1 (2) direction. These two ribbons have the same configuration, so that we only discuss the ribbon of green wireframe. The projected band dispersions are calculated in Figs. S7b and S7c for Hermitian and non-Hermitian cases, respectively. The gray dots are the trivial edge states, and the others are the bulk states. For the non-Hermitian case, we show the degree of localization of each eigenstate by using color, in which the redder (bluer) color represents the more localization at the upper (lower) boundary. The specific value of the color in Fig. S7c is calculated by formula $D = \sum_{x\in L_u}|\psi(x)|^2 - \sum_{x\in L_l}|\psi(x)|^2$, where L_u and L_l are the defined boundary lengths for upper and lower boundary, respectively. The whole ribbon includes 37 sites and each of the upper and lower boundary length contains 10 sites closest to them. So that the skin modes locating on the upper (lower) boundary possess positive (negative) D. One can see that lots of bulk states for $k_1 > 0$ $(k_1 < 0)$ are the skin modes localized at the upper (lower) boundary. Figure S7d shows the concrete examples of the skin and edge modes. Note that the edge state (green star) is only localized at one type of sublattices, which is different from the skin modes (magenta and blue stars). In Fig. S7e, we calculate the spatial distribution $W(j)$ for k_1 from -1 to 1 with interval 0.01, which reveals the skin effect localized at both the upper and lower boundaries.

The skin effect exists in the ribbon under diamond-stripe geometry, but disappears in the ribbon under square-stripe geometry. Therefore, the skin effect is called the GDSE.

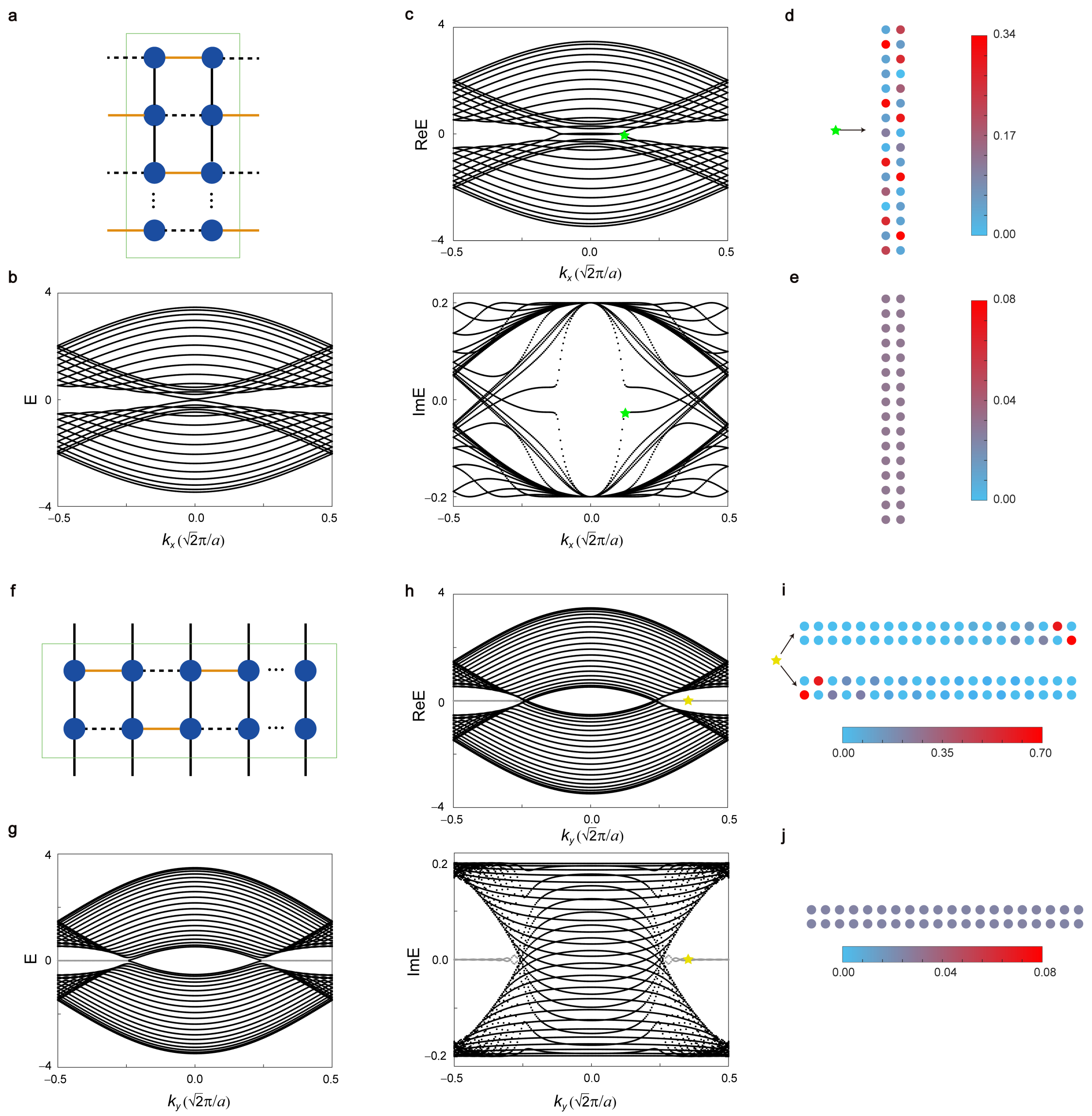

Fig. S6 | No skin effect in the 1D ribbons under square-stripe geometry. a, Schematic of the ribbon with periodic boundaries along the x direction and open boundaries along the y direction. **b**, Projected band dispersion in Hermitian case ($\gamma = 0$). **c**, Projected band dispersion in non-Hermitian case ($\gamma = 0.2$). Top (bottom) panel is the real (imaginary) part. **d**, Field distribution of an eigenstate denoted by green stars in **c**. **e**, Spatial distribution of eigenstate $W(j)$ for the ribbon. **f-j**, The same to **a-e**, but for the ribbon with periodic boundaries along the y direction and open boundaries along the x direction.

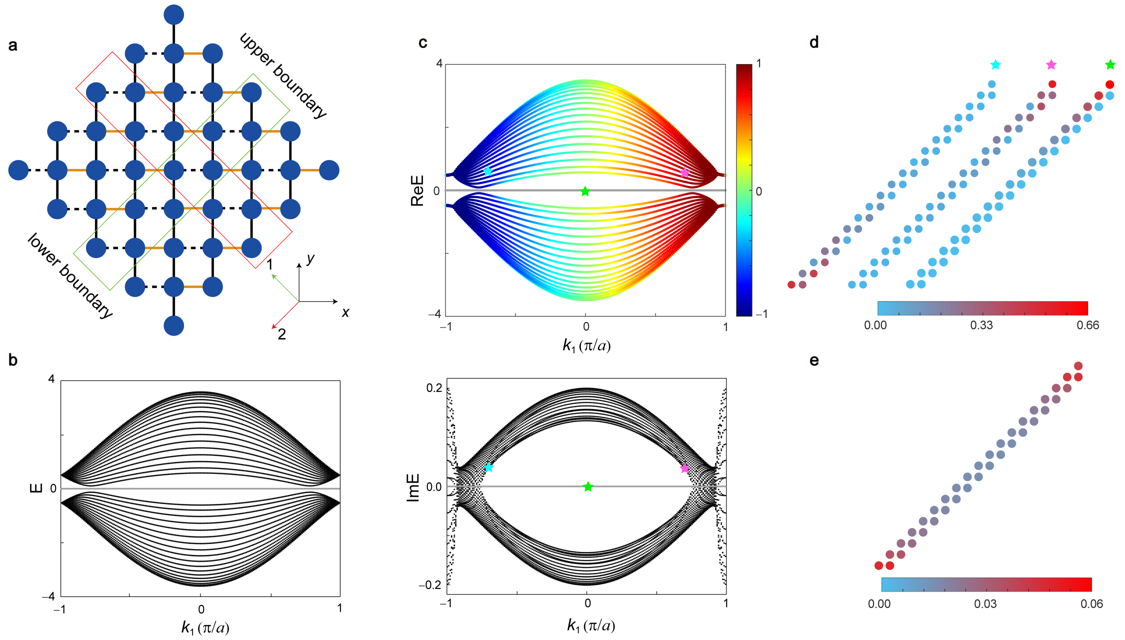

Fig. S7 | Skin effect emerging in the 1D ribbons under diamond-stripe geometry. **a**, Schematic of the ribbons under diamond-shaped geometry. The green (red) wireframe encloses a ribbon with periodic boundaries along k_1 (k_2). **b**, Projected band dispersion in Hermitian case ($\gamma = 0$). Gray dots are the edge states. **c**, Projected band dispersion in non-Hermitian case ($\gamma = 0.2$). Top (bottom) panel is the real (imaginary) part. The color denotes the degree of localization of the eigenstates at the upper and lower boundaries. **d**, Field distributions of three eigenstates denoted by cyan, magenta and green stars in **c**. **e**, Spatial distribution of eigenstates $W(j)$.

H. Difference between square-shaped and diamond-shaped geometries

The fundamental difference between square-shaped and diamond-shaped geometries is that their open boundaries are in different directions, and more importantly, the spectral winding numbers along these directions are different, leading to GDSE, as shown in Fig. S3e. Specifically, the open boundaries of square-shaped geometry are in the x and y directions, and the spectral winding numbers of the straight lines along the k_x and k_y directions are zero, thus no skin effect appears in the sample under square-shaped geometry. However, the open boundaries of diamond-shaped geometry are in the 1 and 2 directions, and the spectral winding numbers

along the k_1 and k_2 directions can be nonzero, guaranteed by the EPs, so the sample under diamond-shaped geometry exhibits the skin effect.

The GDSE is the phenomenon that there is at least one geometry with fully open boundary, in which the skin effect does not appear [1]. According to this definition, the square- and diamond-shaped geometries do be enough to demonstrate GDSE. The other shape can also exhibit the GDSE, such as the sample under parallelogram-shaped geometry with sloping open boundaries possessing slope 2, as calculated in Fig. S5. No skin effect emerges at the horizontal open boundaries, same to the case under square-shaped geometry. But the skin effect appears at the sloping open boundaries, owning to the nonzero winding numbers along the corresponding direction.

I. Calculation of volume law

Different from the skin effect in one dimension and the higher-order skin effect, in which all the bulk modes are the skin modes, not all the bulk modes are the skin modes in the GDSE. However, the GDSE still satisfies the volume law that the number of the skin modes scales with the volume of the system. We define the skin mode with

$$\sum_{x \in S} |\psi(x)|^2 \geq 80\%, \tag{S15}$$

where S is the defined boundary area. To verify the volume law, we choose the parallelogram-shaped geometry with different boundaries shown in Fig. S8a. The sloping open boundaries with slope 1 are the same as the boundaries in the 1 direction under diamond-shaped geometry, while the horizontal open boundaries are the same as the boundaries in the y direction under square-shaped geometry. The area outside the dashed lines is the defined boundary area with the width of four atoms on both sides. As the width of the whole geometry increases, the width of the boundaries also increases proportionally. The bulk Hamiltonian is the same as Eq. (S3) with $t_1 = t_y = -1$, $t_2 = -0.5$, and we discuss two cases with $\gamma = 0.2$ and $\gamma = 1$, respectively. According to the definition of skin modes in Eq. (S15), we obtain the number of skin modes $\mathrm{N_s}$ under different geometry size, as shown in Fig. S8b. The red (blue) straight line is the fitting curve for those data points with $\gamma = 1$ ($\gamma = 0.2$), which shows that the GDSE satisfies volume law, and the relationships between $\mathrm{N_s}$ and volume V

satisfy $\delta N_s \approx 0.45\delta V$ ($\delta N_s \approx 0.17\delta V$).

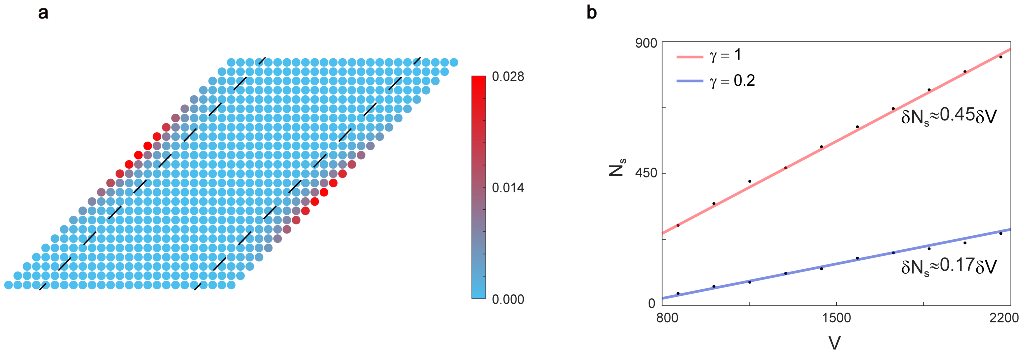

Fig. S8 | The volume law of GDSE. a, Calculated spatial distribution of eigenstates $W(j)$ under parallelogram-shaped geometry with sloping open boundaries possessing slope 1. The area outside two black dashed lines is the defined boundary area. **b**, Volume law of GDSE in our system. The point graph is plotted by calculating data and the red (blue) straight line is the fitting curve showing the relationship $\delta N_s \approx 0.45\delta V$ ($\delta N_s \approx 0.17\delta V$) for $\gamma = 1$ ($\gamma = 0.2$).

S-II. Designed loss in experiments

The designed loss in experiments can be simulated by the imaginary part of sound velocity [4]. A structure with two cavities connected by one waveguide is considered in Fig. S9a, and its sizes are same to those in the main text. Two holes on waveguide not only induce the loss, but also change the coupling strength and give rise to the frequency shift. In order to reduce the frequency shift, sound-absorbing sponges are used to plug in the holes. Moreover, the right amount of sponge will increase the designed loss. We place a point source in one cavity, and measure the response of pressure in another one. The simulated and experimental results of some loss cases are illustrated in Figs. S9b and S9c, respectively, where $i = 0,10,\ldots,50\ \mathrm{m/s}$ is the imaginary velocity of waveguide, and $d = 0,1.6,\ldots,3.2\ \mathrm{mm}$ represents the diameter of hole on waveguide. They are consistent well with each other. In simulation, we add imaginary velocity $(4.3\ \mathrm{m/s})$ on each cavity as global loss existed in experiment. The results for the Hermitian ($i = 0$ and $d = 0$ in Fig. S9d) and non-Hermitian ($i = 50\ \mathrm{m/s}$ and $d = 3.2\ \mathrm{mm}$ in Fig. S9e) cases both show a good agreement in the simulation and

experiment. Consequently, the designed loss in experiment can be estimated by the simulation setting with imaginary velocity $i = 50$ m/s. We note in passing that the two resonant peaks indicate the corresponding eigenmodes, which are split from a dipole mode in a single cavity [5].

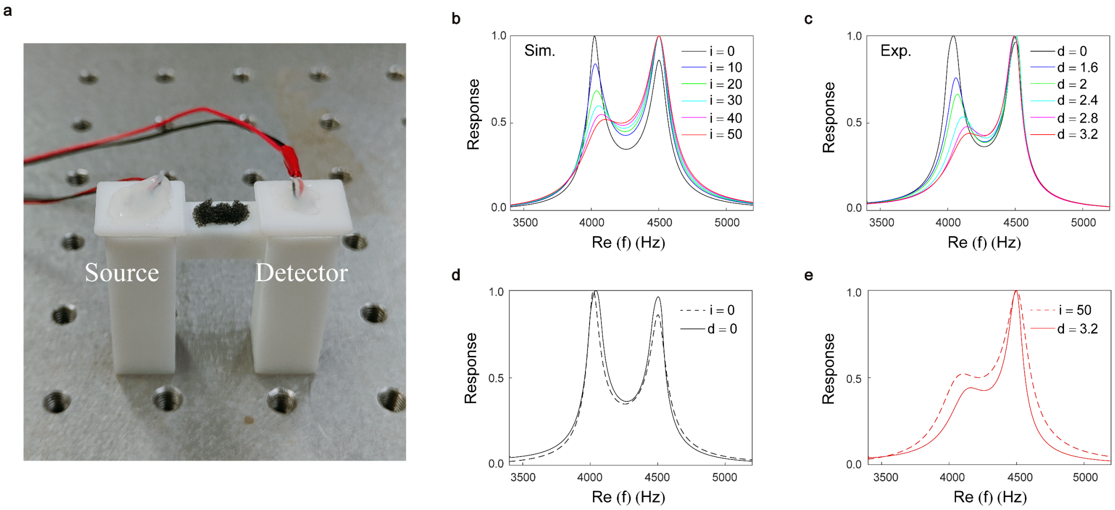

Fig. S9 | Designed loss in experiments. a, Two cavities are connected by waveguide with the designed loss. **b**, Simulated pressure response to different imaginary velocity in waveguide. **c**, Measured pressure response to different diameters of hole on the waveguide. **d**, **e**, Comparisons between the simulated and measured results for the Hermitian (**d**) and non-Hermitian (**e**) cases.

S-III. Detailed spectra of the PC

In this section, we provide the detailed spectra of the PC, as shown in Fig. S10, including the dispersion curves (real and imaginary parts) along high symmetry lines, as a function of k_x for fixed k_y, and as a function of k_y for fixed k_x. These results clearly show the exceptional points and bulk Fermi arcs existed in the PC.

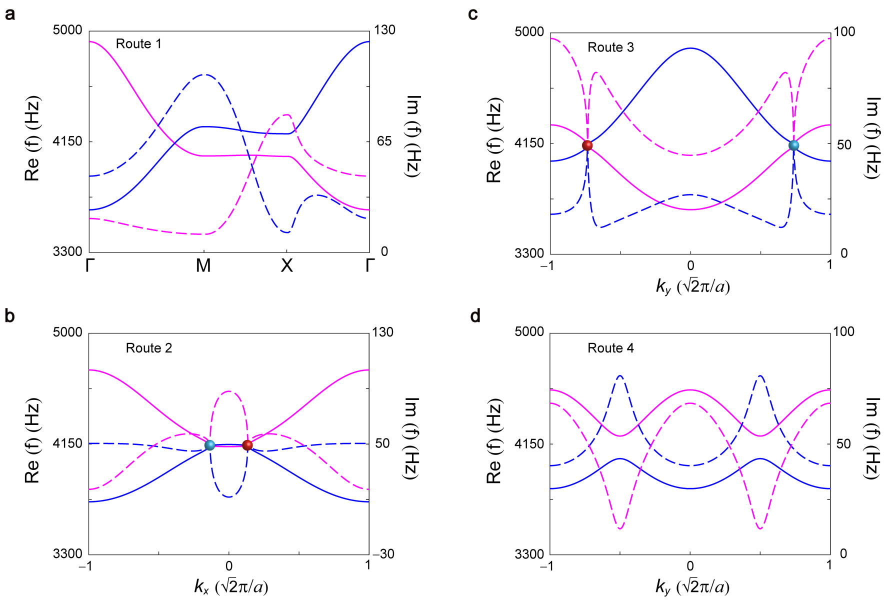

Fig. S10 | Bulk band dispersions of the PC for different routes. a-d, Dispersion curves containing both real (solid lines) and imaginary parts (dashed lines) along the route 1-4 in momentum space shown in Fig. S2b. Degeneracy of the bulk Fermi arc emerges in **a**, while degeneracies of exceptional points occur in **b** and **c**. No degeneracy occurs in **d**.

S-IV. Observation of the bulk Fermi arc in the 2D PC

In this section, we present the observations of the bulk dispersion and bulk Fermi arc in details. The experimental sample has been shown in Fig. 2a of the main text. We place a point source in the center of structure, and measure the response signal of cavities. After the Fourier transform of the response signal, the measured dispersion can be obtained and displayed by the color map. Figures S11a and S11b denote the simulated and measured bulk band dispersions along the high-symmetry lines, where the intersection is one of points on the bulk Fermi arc. To further demonstrate the existence of the bulk Fermi arc, we display the simulated (Figs. S11c, S11e, S11g) and measured (Figs. S11d, S11f, S11h) isofrequency curves in the first BZ at different frequencies near the EP, where magenta lines are the calculated isofrequency contours. One can see that the bulk Fermi arcs are observed at frequency 4150 Hz (Figs. S11e and S11f). All the experimental results are well consistent with the simulated ones.

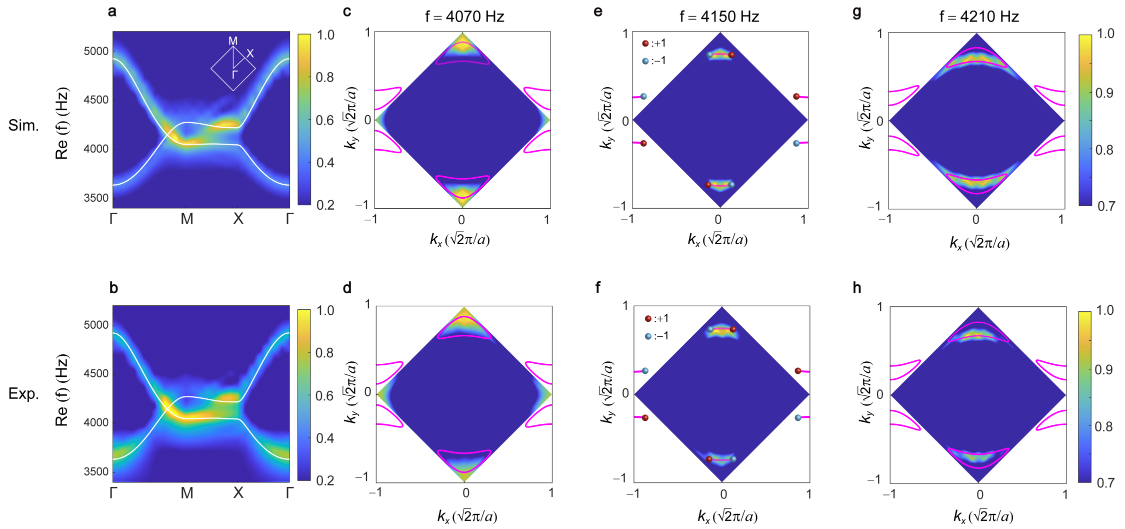

Fig. S11 | Observation of the bulk Fermi arc. **a**, **b**, Simulated and measured bulk band dispersions (color map). White lines are the calculated bulk band dispersion. **c**-**h**, Simulated (**c**, **e**, **g**) and measured (**d**, **f**, **h**) isofrequency curves in the first BZ at different frequencies. Magenta lines are the calculated isofrequency contours. The bulk Fermi arcs appear at 4150 Hz.

S-V. Band dispersions near the EPs for different temperatures

The EPs with topological charge ± 1 are stable in two dimensions. So, the existence of EPs in our system is not sensitive to temperature, but their positions and frequencies may be changed by the temperature. Specifically, the temperature can affect the sound velocity, thereby acts on the band dispersion in the PC. In general, the relation between sound velocity c and temperature T (℃) is estimated by $c = 331\sqrt{1 + T/273}\ \mathrm{m/s}$. When the temperature is confined at 15 ℃, 24 ℃ and 33 ℃, the sound velocity is about 340 $\mathrm{m/s}$, 345 $\mathrm{m/s}$ and 350 $\mathrm{m/s}$, respectively. Figure S12 gives the band dispersions near the EPs for these three temperatures. One can see that the EPs emerge in all these cases, and their frequencies lift as the temperature rises. As a result, the temperature mainly causes a frequency drift, but does not affect the existence of EPs.

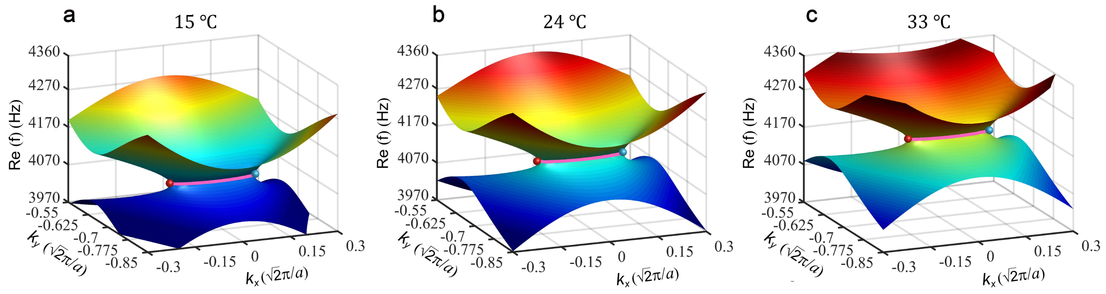

Fig. S12 | Band dispersions near the EPs for different temperatures. a-c, At the temperatures 15 °C, 24 °C and 33 °C, respectively. In our experiment, the temperature is confined at 24 °C.

S-VI. GDSE in the 1D PC ribbons

In this section, we discuss the skin effect in the 1D PC ribbons under diamond-stripe and square-stripe geometries. For the ribbon under diamond-stripe geometry, we calculate the projected band dispersion along the k_1 direction, as shown in Fig. S13a. As discussed in Fig. 3 of the main text, lots of bulk states for $k_1 < 0$ ($k_1 > 0$) are the skin modes localized at the lower (upper) boundary, which is consistent with the theoretical model in Fig. S7. The skin modes and edge modes are visualized by the field distributions shown in Fig. S13b. The spatial distribution $W(j)$ (Fig. S13c) calculated by the method same to Fig. S7e but with interval 0.025 reveals that the skin effect appears at both boundaries, and the edge states have little influence on the skin effect. In contrast, considered a PC ribbon under square-stripe geometry, whose projected band dispersion along the k_x direction is shown in Fig. S13d, we find the bulk modes are not the skin modes, as shown in Fig. S13e. The corresponding spatial distribution $W(j)$ calculated by summing k_x from -0.5 to 0.5 with interval 0.0125 is shown in Fig. S13f. The uniform distribution indicates no skin effect for this case. These results are the direct evidences of GDSE.

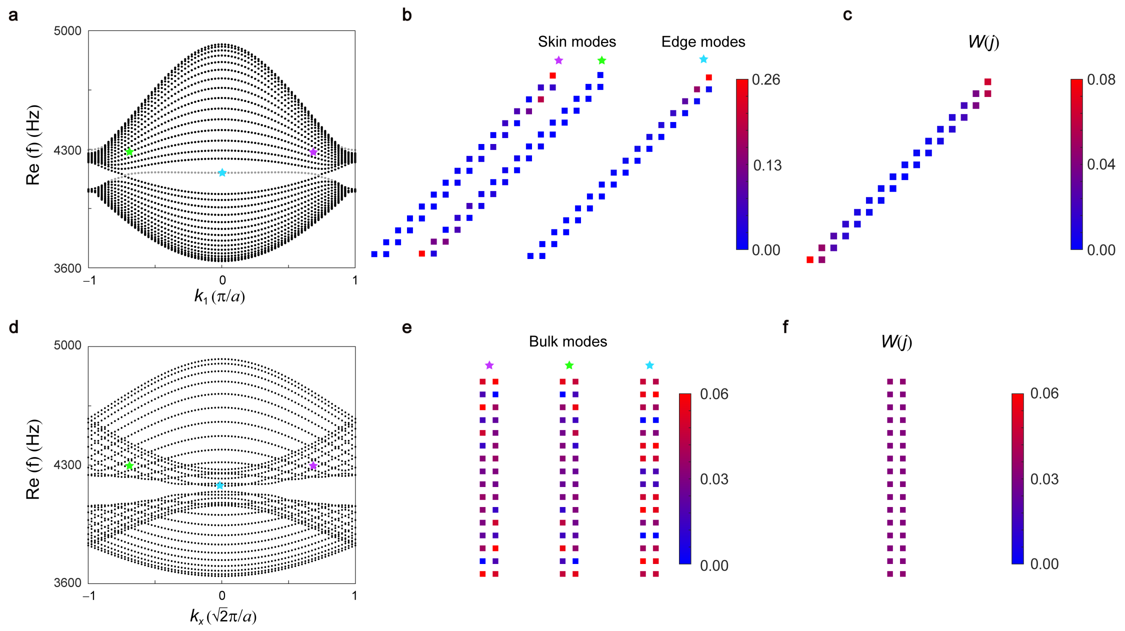

Fig. S13 | GDSE in the 1D PC ribbons. **a**, Real part of projected band dispersion for the PC ribbon under diamond-stripe geometry. The black (grey) dots represent the bulk (edge) dispersions. **b**, Field distributions of three eigenstates denoted by green, cyan, and purple stars in **a**. **c**, Spatial distribution of eigenstates $W(j)$. **d-f**, The same to **a-c**, but for the PC ribbon under square-stripe geometry.

S-VII. No skin effect in the finite-size PC under square-shaped geometry

For comparison with the diamond-shaped PC in the main text, we construct a square-shaped PC, as shown in Fig. S14a. The corresponding spectral area is plotted in Fig. S14b (red dots), where the blue area is the spectral area under periodic boundaries with details shown in Fig. 2h of the main text. Spectral areas and their density distributions under these two boundary conditions coincide with each other, indicating that there is no skin effect in this PC sample. The corresponding real part of eigenfrequency spectrum is shown in Fig. S14c, in which the color denotes the degree of localization for each eigenstate on open boundaries. To keep the similar cavity number of the boundary area, it is defined as the outermost three layers cavities, and the PC sample under square-shaped geometry has 22 cavities at the outermost boundary. One can see that the color is uniform for all the frequency, except the edge modes localized at the left and right sides (inset in Fig. S14c), indicating no skin modes in this

finite-size sample. In experiment, we measure the response spectrum as shown in Fig. S14d, the red line (T_{12}) is the response spectrum on the edge, where the source (detector) is located at point 1 (2) marked in Fig. S14a. Its distribution is similar to it of the response spectrum in the bulk T_{34} (black line). To visualize the specific modes, we further display the measured pressure field distributions at 4003 Hz (bulk mode) and 4160 Hz (trivial edge mode) in Figs. S14e and S14f, respectively. The results confirm no skin effect in the finite-size PC sample under square-shaped geometry.

In theory, the skin effect can be revealed by the spatial distribution of all eigenstates $W(j)$. As calculated in Fig. S15a, the spatial distribution $W(j)$ for the finite-size PC samples under square-shaped geometry is uniform on the geometry, confirming no skin effect. In contrast, the spatial distribution $W(j)$ under diamond-shaped geometry calculated in Fig. S15b has a stronger distribution at the boundaries, proving the existence of skin effect.

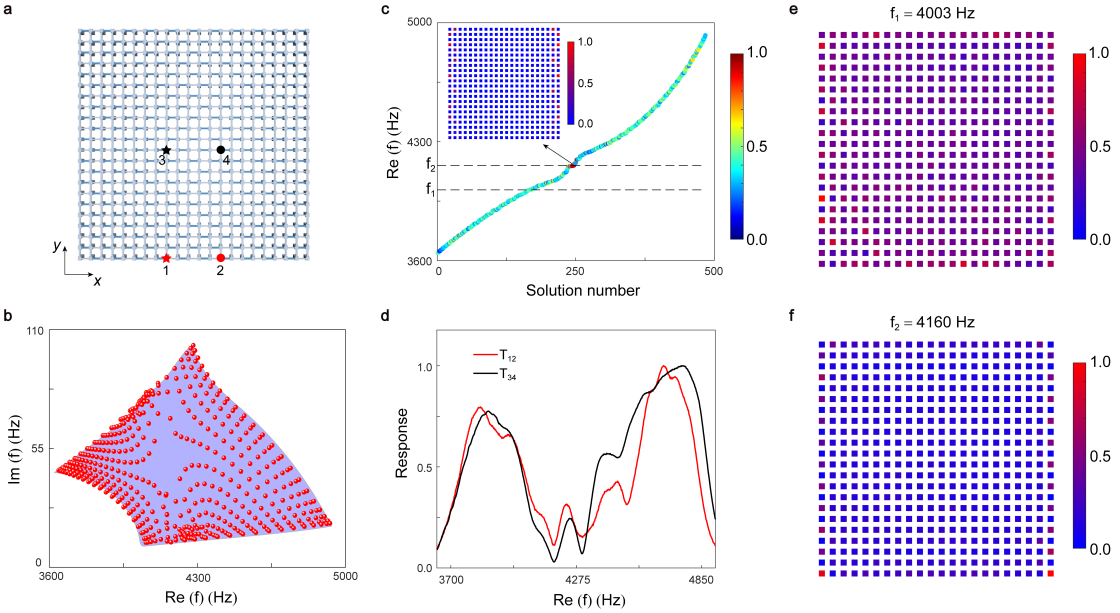

Fig. S14 | No skin effect in the finite-size PC sample under square-shaped geometry. **a**, Schematics of the finite-size PC sample. **b**, Spectral area (red dots) for the square-shaped PC. Blue shadow area denotes the spectrum under periodic boundaries. **c**, Real part of eigenfrequency spectrum. The color represents the degree of localization for each eigenstate on open boundaries. Inset: eigenpressure field of the edge mode. **d**, Measured response spectra at boundary (T_{12}) and bulk (T_{34}) normalized by their

maximum values, which both show the bulk modes. **e**, **f**, Measured pressure field distributions at 4003 Hz and 4160 Hz, revealing the bulk and edge modes, respectively. No skin modes are observed in the square-shaped PC.

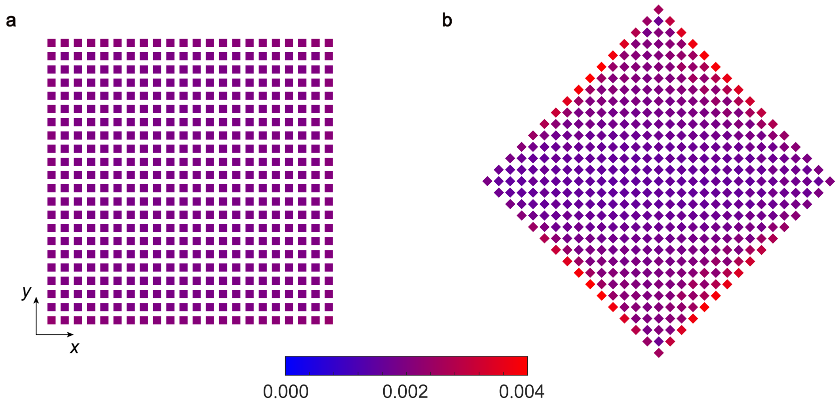

Fig. S15 | Spatial distribution of all eigenstates $W(j)$ in the finite-size PC samples. **a**, $W(j)$ for the sample under square-shape geometry. **b**, $W(j)$ for the sample under diamond-shaped geometry.

S-VIII. Pressure responses for different distances away from source

In this section, we display the pressure for different distances away from source. As shown in Fig. S16a, the signal can be detected in all the cavities P_1-P_3, so the detectable distance of an excited field in Fig. 3 of the main text is 8 cavities at least. While in Fig. 4 of the main text, the signal can be detected in the cavities P_4 and P_5 for all concerned frequencies, but in the cavity P_6 only for the higher frequencies, as shown in Fig. S16b, so the detectable distance is more than 8 cavities but less than 15 cavities.

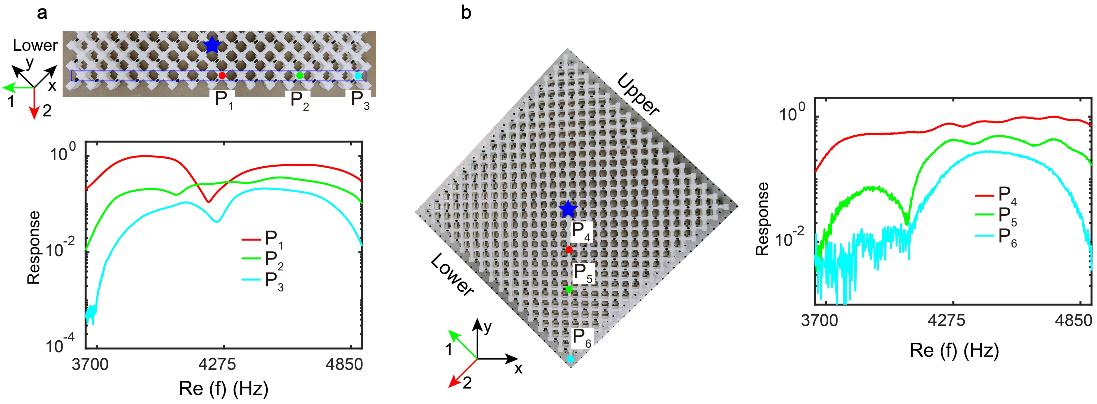

Fig. S16 | Observation of the pressure responses for different distances away from source. a, Photograph of lower boundary (upper panel) and the measured pressures at P_1, P_2 and P_3 cavities (lower panel). **b**, Photograph of diamond-shaped sample (left panel) and the measured pressures at P_4, P_5 and P_6 cavities (right panel).

References

[1] Zhang, K., Yang, Z. & Fang, C. Universal non-Hermitian skin effect in two and higher dimensions. Nat. Commun. **13**, 2496 (2022).

[2] Kawabata, K., Shiozaki, K., Ueda, M. & Sato, M. Symmetry and topology in non-Hermitian physics. Phys. Rev. X **9**, 041015 (2019).

[3] Gong, Z. P., Ashida, Y., Kawabata, K., Takasan, K., Higashikawa, S. & Ueda, M. Topological phases of non-Hermitian systems. Phys. Rev. X **8**, 031079 (2018).

[4] Gao, H., Xue, H., Gu, Z., Liu, T., Zhu, J. & Zhang, B. Non-Hermitian route to higher-order topology in an acoustic crystal. Nat. Commun. **12**, 1888 (2021).

[5] Qi, Y., Qiu, C., Xiao, M., He, H., Ke, M. & Liu, Z. Acoustic realization of quadrupole topological insulators. Phys. Rev. Lett. **124**, 206601 (2020).
